# Supplementary material for: Post-Resuscitation Partial Pressure of Arterial Carbon Dioxide and Outcome in Patients with Out-of-Hospital Cardiac Arrest: A Multicenter Retrospective Cohort Study
Source: J Clin Med. 2022 Mar 10;11(6):1523. doi: 10.3390/jcm11061523 (PMC8954853; doi:10.3390/jcm11061523)
Supplement: Supplementary file 1 [file jcm-11-01523-s001.zip › jcm-1598368-supplementary.pdf]

Supplementary Materials

Supplementary Table S1. Outcomes for the patient population excluding patient with treatment using a mechanical circulatory device.

(A) Association with 1-month poor neurologic status and exposure to PaCO2 of 24 hours post-return of spontaneous circulation.

	Total	1-month poor neurologic status[a] (%)		Crude OR	(95% CI)	Adjusted[b] OR	(95% CI)
Severe hypocapnia exposure	32	29	(90.6)	12.0	(3.38-42.3)	42.3	(4.37-409.35)
Mild hypocapnia exposure	154	100	(64.9)	2.29	(1.33-3.93)	3.00	(1.34-6.75)
Normocapnia exposure	78	39	(50.0)	1.24	(0.67-2.29)	2.47	(0.98-6.21)
Mild hypercapnia exposure	85	38	(44.7)	Reference		Reference	
Severe hypercapnia exposure	86	65	(75.6)	3.82	(1.99-7.35)	3.59	(1.34-9.65)
Both exposure	58	45	(77.6)	4.28	(2.02-9.07)	10.35	(3.40-31.48)

(B) Association with 1-month mortality and exposure to PaCO2 of 24 hours post-return of spontaneous circulation.

	Total	1-month mortality (%)		Crude OR	(95% CI)	Adjusted[b] OR	(95% CI)
Severe hypocapnia exposure	32	17	(53.1)	3.68	(1.56-8.67)	2.46	(0.74-8.15)
Mild hypocapnia exposure	154	53	(34.4)	1.71	(0.93-3.11)	1.76	(0.77-4.01)
Normocapnia exposure	78	21	(26.9)	1.20	(0.59-2.43)	2.22	(0.83-5.92)
Mild hypercapnia exposure	85	20	(23.5)	Reference		Reference	
Severe hypercapnia exposure	86	36	(41.9)	2.34	(1.21-4.52)	1.66	(0.67-4.13)
Both exposure	58	29	(50.0)	3.25	(1.58-6.67)	4.53	(1.71-12.0)

CI, confidence interval; OR, odds ratio.

[a]Poor neurologic status defined as Cerebral Performance Category≥ 3

[b]Adjusted for sex, age, witnessed arrest, bystander performed cardiopulmonary resuscitation, initial cardiac rhythm, cardiopulmonary resuscitation duration >10 min, Glasgow Coma Score at hospital arrival, hyperoxia exposure, targeted temperature management, cause of cardiac arrest, $PaCO_2$ immediately after return of spontaneous circulation, $PaCO_2$ group.
